# Challenges of Inversely Estimating Jacobian from Metabolomics Data

**DOI:** 10.3389/fbioe.2015.00188

**Published:** 2015-11-18

**Authors:** Xiaoliang Sun, Bettina Länger, Wolfram Weckwerth

**Affiliations:** ^1^Department of Ecogenomics and Systems Biology, University of Vienna, Vienna, Austria; ^2^Institute of Integrative Biology, University of Liverpool, Liverpool, United Kingdom; ^3^Vienna Metabolomics Center (VIME), University of Vienna, Vienna, Austria

**Keywords:** metabolomics, inverse engineering, Lyapunov Equation, Jacobian, ill-posed problems

## Abstract

Inferring dynamics of metabolic networks directly from metabolomics data provides a promising way to elucidate the underlying mechanisms of biological systems, as reported in our previous studies (Weckwerth, 2011; Sun and Weckwerth, 2012; Nägele et al., 2014) by a differential Jacobian approach. The Jacobian is solved from an overdetermined system of equations as *JC* + *CJ^T^* = −2*D*, called Lyapunov Equation in its generic form,^1^ where *J* is the Jacobian, *C* is the covariance matrix of metabolomics data, and *D* is the fluctuation matrix. Lyapunov Equation can be further simplified as the linear form *Ax* = *b*. Frequently, this linear equation system is ill-conditioned, i.e., a small variation in the right side *b* results in a big change in the solution *x*, thus making the solution unstable and error-prone. At the same time, inaccurate estimation of covariance matrix and uncertainties in the fluctuation matrix bring biases to the solution *x*. Here, we first reviewed common approaches to circumvent the ill-conditioned problems, including total least squares, Tikhonov regularization, and truncated singular value decomposition. Then, we benchmarked these methods on several *in silico* kinetic models with small to large perturbations on the covariance and fluctuation matrices. The results identified that the accuracy of the reverse Jacobian is mainly dependent on the condition number of *A*, the perturbation amplitude of *C*, and the stiffness of the kinetic models. Our research contributes a systematical comparison of methods to inversely solve Jacobian from metabolomics data.

## Introduction

Understanding regulatory mechanisms of metabolic networks at the systems level is a demanding, yet essential task. Metabolomics is the study of all metabolites identified and quantified in a biological organism under a specified physiological state and provides a promising approach to potentially unravel the complex dynamics in metabolic systems by measuring many metabolites participating in particular biochemical processes and across many biological samples (Nicholson et al., 1999; Fiehn et al., 2000; Weckwerth, 2003; Weckwerth et al., 2004). One central goal in applying these technologies is to study how metabolic networks respond to different treatments, such as environmental stresses, genetic mutations. Because metabolic networks typically consist of many non-linear interactions (Strogatz, 1994) between metabolites, identifying perturbation sites from metabolomics data is one of the major challenges. Theoretical frameworks have been introduced to detect perturbation sites and to understand dynamic features of metabolic networks. Current approaches to the analysis of experimental data can be divided into three categories: statistical analysis, dynamic modeling, and network analysis. Multivariate statistical methods, such as principal and independent components analysis (Nicholson et al., 1999; Fiehn et al., 2000; Raamsdonk et al., 2001; Morgenthal et al., 2005), correlation network analysis (Weckwerth, 2003; Weckwerth et al., 2004; Camacho et al., 2005), clustering analysis (Roessner et al., 2001), partial least squares discrimination analysis (Bijlsma et al., 2006), support vector machines (Zhang et al., 2006), and many others [for a comprehensive review, see Sugimoto et al. (2012)] aim at analyzing the complex relationships between the measured molecules and to reveal the inherent data structure in order to find associations between the different molecules and, eventually, causality to infer the directionality of metabolic and regulatory processes. Though powerful in classifying samples and providing insights into cellular activities under different treatment conditions, they lack the ability to detect perturbation sites associated with the dynamics of the underlying metabolic reaction system.

As a more analytical approach, mathematical modeling represents metabolic networks as a set of ordinary differential equations (ODEs, Eq. 1) where *S*_1_, *S*_2_, … , *S_n_* are the concentration of *n* metabolites and *f*_1_, *f*_2_, … , *f_n_* are the rate of enzymatic reactions, such as Michaelis–Menten kinetics or mass action.

(1) $$\frac{\mathrm{df}}{\mathrm{dt}}=\frac{\partial f}{\partial S}\frac{\partial S}{\partial t}=J\left.\left\{\begin{array}{c} \frac{dS_{1}}{\mathrm{dt}}=f_{1}\left(S_{1},S_{2},\cdots ,S_{n}\right)\\ \frac{dS_{2}}{\mathrm{dt}}=f_{2}\left(S_{1},S_{2},\cdots ,S_{n}\right)\\ \vdots \\ \frac{dS_{n}}{\mathrm{dt}}=f_{n}\left(S_{1},S_{2},\cdots ,S_{n}\right) \end{array}\right.\right\}$$

The Jacobian matrix *J* (Eqs 1 and 2) is the first-order derivative of the reaction rate *f_i_* (*i* = 1, 2, … , *n*) with respect to the concentration of metabolites *S_j_* (*j* = 1, 2, … , *n*). It describes the influence on the change of each metabolite upon the changes of other metabolites, and thus captures the reaction-level changes under perturbations, such as environmental stress and gene mutations, to the metabolic network. Therefore, the Jacobian matrix is very useful to understand regulatory mechanisms of metabolic networks at the systems level.

(2) $$\text{Jacobian}=\frac{\partial f}{\partial S}={\left(\begin{array}{cccc} \frac{\partial f_{1}}{\partial S_{1}} & \frac{\partial f_{1}}{\partial S_{2}} & \cdots & \frac{\partial f_{1}}{\partial S_{n}}\\ \frac{\partial f_{2}}{\partial S_{1}} & \frac{\partial f_{2}}{\partial S_{2}} & \cdots & \frac{\partial f_{2}}{\partial S_{n}}\\ \vdots & \vdots & \ddots & \vdots \\ \frac{\partial f_{n}}{\partial S_{1}} & \frac{\partial f_{n}}{\partial S_{2}} & \cdots & \frac{\partial f_{n}}{\partial S_{n}} \end{array}\right)}_{n\times n}$$

To obtain the Jacobian matrix, it is natural to build mathematical models (such as Eq. 1) from metabolomics data. However, there are several practical difficulties. Firstly, kinetic parameters of reaction rate *f* are unknown. If these parameters are not easily obtained by enzymatic assays, parameter estimation should be used, but it is not a trivial work. It involves literature mining: parameters are collected from different individual studies where they can range over several orders of magnitude. Tuning these parameters to validate the model, i.e., minimize the differences between model simulation results and experimental data, is usually a long and iterative process, and cannot achieve a satisfactory result (Gutenkunst et al., 2007). As the result, most metabolic models are limited to a small scale, ranging from several to a few dozens of metabolites. To our knowledge, there are no metabolomics-scale kinetic models. Secondly, the detailed types of kinetics of some reactions may not be known (Goel et al., 2008), for example, whether the kinetics of one reaction is mass action or Michaelis–Menten type. Estimation of the equation forms may even be more difficult than parameter estimation. Thirdly, metabolic processes are full of fluctuations that may result from stochastic transcription factor activities, cross-membrane translocation noise, and cross-talk between reactions or pathways (Rao et al., 2002; Paulsson, 2005; Raser and O’Shea, 2005). On the phenotypic level, it was demonstrated in a metabolomics study that the variations between biological samples are too large to be explained by technical errors (Morgenthal et al., 2006). From this perspective, ODE-based deterministic modeling is not able to reflect these variations. Stochastic modeling is needed. However, the numeric methods to solve stochastic differential equations (SDEs) are not yet well established, in particular, no efficient methods are existing for big and stiff systems, such as the metabolomics-scale system.

Steuer et al. established a fundamental link between metabolic covariance data *C* and the Jacobian matrix *J* by expanding the Lyapunov Equation (Eq. 3) where the right side *D* is the fluctuation matrix in which the diagonal entries characterize the fluctuation magnitude of each metabolite. *J^T^* is the transposed form of *J* (Steuer et al., 2003).

(3) $$\mathrm{JC}+CJ^{T}=-2D$$

For a system with *n* metabolites, there are *n*^⋆^(*n* + 1)/2 independent values in the symmetric covariance matrix *C* but *n*^2^ variables in the non-symmetric *J* to be determined. In other words, the number of equations is smaller than the number of variables, thus Eq. 3 is underdetermined. Most underdetermined systems have no unique solutions. The authors suggested using parameterized solutions to eliminate such underdetermination. However, as the parameter space for uncertain parameters is large, the actual Jacobian may not easily be obtained by such parameterization.

We can circumvent this problem by introducing the stoichiometric matrix (STOI) of a metabolic network, which is typically very sparse (Weckwerth, 2011; Sun and Weckwerth, 2012). If STOI and the reversibility of reactions can be determined, then it is possible to determine non-zero entries in the Jacobian *J*. Fortunately, the information for the reversible and irreversible reactions can be obtained by genome-scale network reconstruction (Weckwerth, 2011) and also based on public accessible database, such as KEGG (Kanehisa et al., 2014) and BioCyc (Caspi et al., 2014). Since metabolic networks are usually very sparse (Sun and Weckwerth, 2012), many entries in *J* are 0s, and consequently, Eq. 3 becomes overdetermined. However, under some circumstances, such as allosteric inhibition, regulation between metabolites is reflected in *J* but not in the STOI. For such cases, we need additional knowledge from literature and databases to assign these non-zero entries in *J*.

Overdetermined systems have best approximation solutions. To make it clearer to understand, with simple matrix operations, Eq. 3 can be converted to the linear form as *Ax* = *b*, where *A* is an *n*^2^*-by-n*^2^ matrix derived from *C*, *x* is an *n*^2^-*by*-*1* vectorized Jacobian matrix *J*, and *b* is an *n*^2^-*by*-*1* vectorized fluctuation matrix *D*. If *p* entries in *J* are not 0s, the size of *A* is eliminated to *n*^2^*-by-p*; *x* and *b* are *p-by-1* vectors. For simplicity, we assume that A has full column rank, i.e., the rank of *A* is *p*.

The most popular method is ordinary least squares (OLS). It minimizes the squared residual error of *Ax* − *b* (Eq. 4).

(4) $$\min||\mathrm{Ax}-b|{|}_{2}$$

The solution *x* is then obtained by Eq. 5, where *A^T^* is the transposed form of *A*.

(5) $$x={\left(A^{T}A\right)}^{-1}A^{T}b$$

However, in some cases, when *A^T^A* is close to singular, its inverse form (*A^T^A*)^−1^ cannot be stably obtained, resulting in inaccurate solutions *x*. To illustrate this problem, we use the singular value decomposition of matrix *A* (Eq. 6.1), where *U* and *V* are orthogonal matrices; Σ is a diagonal matrix with diagonal elements σ_1_ ≥ σ_2_ ≥ ,…, ≥ σ*_n_*, which are singular values of *A*; their squared form σ^2^ are the Eigen values of matrix *A^T^A*. By definition, for the *i*th singular value σ*_i_*, Eqs 6.2 and 6.3 are obtained. If, for example, σ*_i_* is very small compared to σ*_1_*, the left side of Eq. 6.3 is then very small, thus *A* is nearly rank deficient, introducing the so-called “ill-posed” numeric problems in Eq. 5.

(6.1) $$A=U\Sigma V^{T}$$

(6.2) $$A\nu_{i}=\sigma_{i}u_{i}$$

(6.3) $$||A\nu_{i}||=\sigma_{i}$$

The metric for ill-posed problems, condition number of *A*, is defined as the ratio of the largest singular value to the smallest singular value, i.e., σ_1_/σ*_n_*. When the condition number is large, Eq. 5 tends to be ill-posed.

One method to alleviate ill-posed problems is to truncate *t* (*t* < *n*) smallest singular values [truncated singular value decomposition (TSVD)] and the corresponding columns and rows in the matrix *U* and *V*, respectively, as Eq. 7, where first *n* − *t* singular values are kept. The new solution is a close approximation of *x* but with increased numerical stability.

(7) $$\min||A_{t}x-b|{|}_{2}$$

A similar method is truncated total least squares (TTLS). Unlike the original truncated SVD form Eq. 6, it implements SVD on the combined matrix [*A*|*b*], and truncates smaller singular values as Eq. 7 does. If we rewrite Eq. 4 as Eq. 8.1 and derive Eq. 8.2 from the combined matrix [*A* | *b*], we can see that TLS solution is robust to perturbations δ*A* on matrix *A*.

(8.1) $$\min||\delta b|{|}_{2}\;\text{subject to}\;\mathrm{Ax}=b+\delta b$$

(8.2) $$\min||\delta A\delta b|{|}_{2}\;\text{subject to (}A+\delta A)x=b+\delta b$$

Another method is called “regularization,” which adds a penalty form in the Eq. 4 as

(9) $$\min\left(||\mathrm{Ax}-b|{|}_{2}+||\Gamma \left(x-x_{0}\right)|{|}_{m}\right)$$

*x*_0_ is the initial estimation of *x*; when *x*_0_ is unknown, it is just 0s. Γ is a function of *x* which puts an *L*-*m* norm constraint on its value. In the simplest form, Γ is multiple of the identity matrix *I* and Eq. 9 becomes Eq. 10, where λ is the sole tuning parameter of regularization. Popular methods determining λ values include L-curve criterion (Hansen, 1992) and cross-validation (Hastie et al., 2001); both obey the rules of bias-variance tradeoff (Hastie et al., 2001).

(10) $$\min\left(||\mathrm{Ax}-b|{|}_{2}+\lambda ||\left(x-x_{0}\right)|{|}_{m}\right)$$

Regarding with *m*, when *m* is 1, the penalty form |*x* − *x*_0_| is the absolute least distance between *x* and *x*_0_, and Eq. 9 is also called LASSO in statistics literature; when *m* is 2, the penalty form denotes the squared Euclidean distance between *x* and *x*_0_, and Eq. 9 is called Tikhonov regularization (TIKH) or Ridge Regression. When *m* is between 1 and 2, Eq. 9 has the name “elastic net.” Both LASSO and elastic net implement variable shrinkage on *x* (shrink some *x* entries to 0s), thus are not desirable in our approach solving the Jacobian entries because the 0 entries have been determined by using the stoichiometric matrix. *m* < 1 or *m* > 2 are rarely used.

So far, we have introduced methods to solve the inverse Jacobian from metabolomics covariance data. In our previous work, we established reverse Jacobian calculation pipeline and implemented OLS, TLS, and TIKH in the software COVAIN (Sun and Weckwerth, 2012), which provides an easy-to-use graphical user interface, detailed manual and example data; thus, biologists can obtain a clear understanding of our approaches. COVAIN can be freely downloaded from our website: http://www.univie.ac.at/mosys/software.html.

We applied our approaches on a real metabolomics dataset (Nägele et al., 2014). The inverse Jacobian identified the significant change of activities of pyruvate dehydrogenase complex which interconverts pyruvic acids, and further experiments validated this change.

However, “no free lunch theorem in optimization” also holds true for these inverse methods since they involve the optimization process. It is possible that some methods perform better than others under specified conditions and for some types of data, and therefore, understanding the factors that affect the performance of the inverse methods is important. Additionally, two practical challenges relate with covariance matrix and fluctuation matrix. Firstly, estimation of the covariance matrix is often problematic due to missing values and outliers in the measurements. Post-experimental data processing, for instance, missing value imputation and outliers adjustment, further exert perturbations to the original covariance matrix, i.e., the ideal “true” one with no missing values or outliers. Secondly, the fluctuation matrix can be retrieved from prior biological knowledge, for example, fluctuation only associates with few particular metabolite(s), or with all metabolites, but such information may not be an accurate reflection of the “true” fluctuation in biological organisms. Therefore, for both cases, it is reasonable to check how such uncertainties affect the reverse Jacobian.

## Materials and Methods

**Figure 1:**
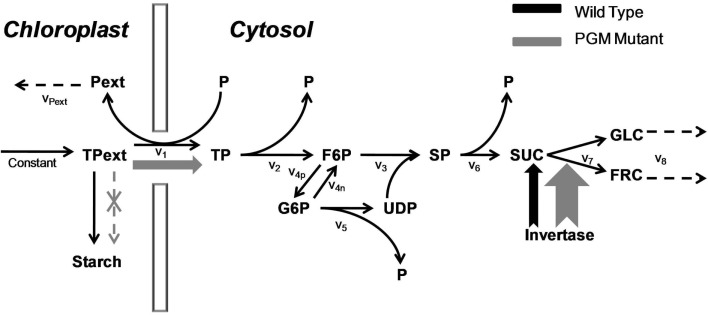
**The sucrose synthesis pathway under wild type and PGM mutant**. The triose phosphate is imported from the chloroplast to synthesize sucrose in the cytosol. The total amount of triose phosphate in the chloroplast is a constant value. In the wide type, most triose phosphate is used to form the starch; in PGM mutant, the starch synthesis pathway is cut off, which results in a much higher triose phosphate flux into the cytosol; at the same time, the activity of invertase is increased. Both increases are shown in wider arrows in gray. The v_1_, v_2_, etc., are the reaction rates with simple mass action kinetics. Abbreviation: Pext, “external” phosphate in the chloroplast; TPext, “external” triose phosphate in the chloroplast; P, phosphate; TP, triose phosphate; F6P, fructose-6-phosphate; G6P, glucose-6-phosphate; UDP, uridine diphosphoglucose – glucose; SP, sucrose phosphate; SUC, sucrose; GLC, glucose; FRC, fructose.

Since our aim is to study the effects of a large condition number, the imperfect covariance matrix and uncertain fluctuation matrix, we choose to use experimentally validated *in silico* models as they are more amenable to introduce perturbations on covariance and fluctuation matrices. The principle of model selection is to select models with different levels of complexity denoted by their sizes and kinetics. We chose one in-house model, the sucrose synthesis model under wild type and PGM-mutant condition in the plant *Arabidopsis thaliana* (Morgenthal et al., 2005) with 11 metabolites and mass action kinetics (abbreviated as Sucrose PGM, Figure 1) and three publicly accessible metabolic models from BioModels database (Le Novère et al., 2006). These three ODEs-based models are:

(1) BIOMD0000000023 (abbreviated as Sucrose BM23, http://www.ebi.ac.uk/biomodels-main/BIOMD0000000023), sucrose accumulation model in the plant *Saccharum officinarum* which contains five metabolites with Michaelis– Menten kinetics;
(2) BIOMD0000000042 (Glycolysis BM42, http://www.ebi.ac.uk/biomodels-main/BIOMD0000000042), glycolysis model in the yeast *Saccharomyces cerevisiae* with 15 metabolites and mostly mass action kinetics and a few complex forms;
(3) BIOMD0000000066 (Signaling BM66, http://www.ebi.ac.uk/biomodels-main/BIOMD0000000066), threonine synthesis model in the bacteria *Escherichia coli* (strain K12) with 11 metabolites and Michaelis–Menten kinetics.

The detailed information of these three models including original publications, kinetic equations, and parameters can be accessed from the BioModels database (Le Novère et al., 2006) in the Systems Biology Makeup Language (SBML) format. We use the default kinetic parameters from the BioModels database. Note that from SBML portal website, http://sbml.org/Documents/FAQ#What_is_this_0.22boundary_condition.22_business.3F, it is recommended not to include constant metabolites in ODE models that are labeled as *boundaryCondition* = “*true”* in the SBML file. For example, for BM23, among 13 metabolites, eight are labeled as constant (these metabolites are Sucvac, glycolysis, phos, UDP, ADP, ATP, Glcex, and Fruex), and we include the rest five in our approach (they are Fru, Glc, HexP, Suc6P, and Suc).

The overall workflow is as follows. We first obtained the *in silico* metabolomics covariance data and Jacobian as well as stoichiometric matrix by simulating the above models in the unperturbed “control” condition with a predefined fluctuation matrix (see below). Second, we introduced different levels of perturbations to the covariance and the fluctuation matrix. Finally, we tested the performance of the inverse Jacobian methods (as shown before) on the perturbed data.

To obtain the metabolomics covariance data, first, we converted the ODEs of above models to SDEs by adding Gaussian white noise to the right side of Eq. 1. Second, we defined the fluctuation matrix *D*_0_ in the control condition as a diagonal matrix (diagonal entries are non-zero and all off-diagonal entries are 0s which means there are no cross-talks between metabolites). Third, we iteratively simulated the SDEs with the predefined *D*_0_ for *N* times and obtained the metabolomics covariance data *C*_0_ and Jacobian *J_0_* in the control condition. For simplicity, we used arbitrary units instead of the real units, but operation on real physical units is straight forward. Chemical reactions, like Eq. 1, have the units of mol L^−1^ s^−1^ or mmol mL^−1^ s^−1^, meaning the concentration change per second. After partial derivation on the concentration variables *S*, the Jacobian *J* (Eq. 2) has units of s^−1^, that is, the inverse of time. The covariance matrix *C* has the units of the squared form of that in the concentration variables, i.e., (mol L^−1^)^2^ or (mmol mL^−1^)^2^.

The perturbation on *C*_0_ was obtained by reducing the repeat times to *N*/2, *N*/3, *N*/10, …. These new covariance matrices *C*_1_, *C*_2_, *C*_3_, etc., thus represent imperfect estimation of *C*_0_, based on the “Law of large numbers” theorem that the covariance estimated from a subset of data does not give the actual approximation of the covariance calculated from the original data. The perturbation magnitude δ*C* is measured by the relative changes to *C*_0_, i.e., $\frac{||C_{i}-C_{0}||}{||C_{0}||}$ (*i* = 1, 2, 3, …).

The perturbed *D*_0_ was achieved by adding different levels of Gaussian white noise to all entries of *D*_0_ as *D_i_* = (*I* + *N*(0, σ^2^))*D*_0_ where *I* denotes the identity matrix and σ is the level of noise. We tested three levels of σ as 0.01, 0.1, and 1. When σ is 0.01, the perturbation magnitudes δ*D*, the relative changes of *D*_0_, $\frac{||D_{i}-D_{0}||}{||D_{0}||}$ (*i* = 1, 2, 3, …), are very small; when σ is 0.1, the magnitudes are observable, and when σ is 1, the new *D_i_* is in fact a fully randomized matrix, where all diagonal and off-diagonal entries have similar amplitude. For each perturbation level of *C*_0_ and *D*_0_, 100 repeats were obtained.

In the inverse Jacobian calculation procedure, we use these perturbed covariance *C_i_* and fluctuation matrices *D_i_* to inversely infer the Jacobian *J_i_* (*i* = 1, 2, 3, …) with the methods introduced above (OLS, TLS, TIKH, and TSVD). The goodness of *J_i_* is represented by the *R*^2^ values of linear regression between *J_0_* and *J_i_*. A limitation of *R*^2^ for linear regression is that they often contain a constant offset from the origin point, and if that happens with the reverse Jacobian approach, it means that entries of *J_0_* and *J_i_* have same “trend,” yet neither comparable nor proportional, and the signs of *J_0_* and *J_i_* entries may be different. However, we showed that both *J_0_* and *J_i_* are crossing the origin point for all models, and thus *J_0_* and *J_i_* entries can be compared in pairwise; therefore, *R*^2^ is a good metric of the goodness of the reverse Jacobian (Figures S1–S4 in Supplementary Material).

## Results

### Condition Number of the Models with Different Perturbation Levels on the Covariance

**Figure 2:**
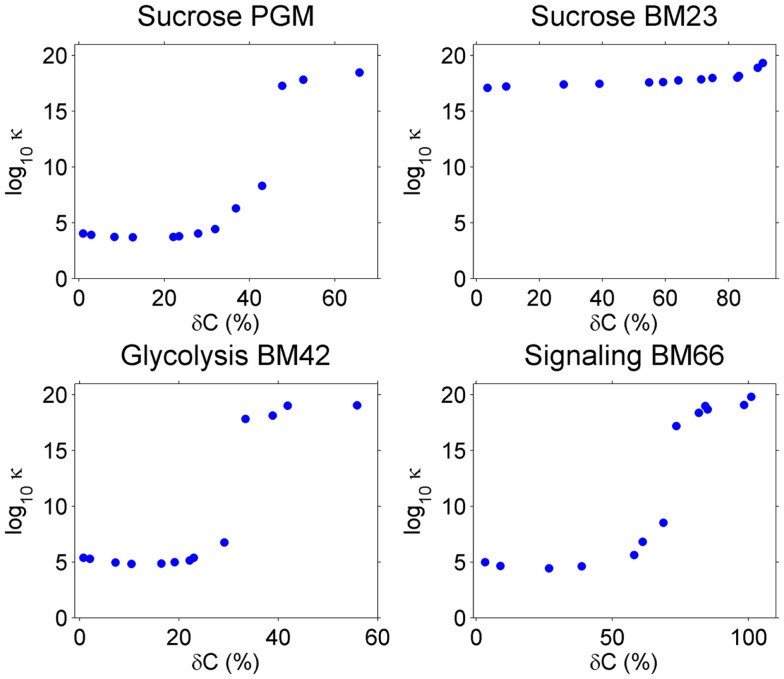
**The condition number κ*_A_* (*y*-axis) increase with higher perturbation amplitude (*x*-axis) on the covariance C**. Note that *y*-axis is in log_10_ scale.

As explained in Section “Introduction,” the condition number of *A*, κ*_A_*, in the linear equations *Ax* = *b* indicates the accuracy of the solution *x* in the overdetermined system. *A* is a function of the covariance *C*, and when perturbations are introduced in *C*, κ*_A_* will be changed. We calculated κ*_A_* for the four models under different perturbation levels on *C* and averaged κ*_A_* over 100 repeats for each perturbation level. Results are shown in Figure 2.

Without perturbation, i.e., δ*C* = 0, the Sucrose PGM model has the lowest condition number (around 10^3^–10^4^), which may be a result of its simple mass action kinetics. Sucrose BM23, on the other side, shows a surprisingly high condition number (over 10^16^), which may result from its complex kinetics. In fact, for this small model with only five metabolites, there are 11 reactions including bireactant Michaelis–Menten kinetics and inhibition regulation, as well as 63 kinetic parameters. Higher complexity of the model may lead to increased fluctuation propagation and result in larger variance–covariance matrices. The other two models, Glycolysis BM42 and Signaling BM66, which contain more metabolites and reactions than the Sucrose PGM model and simpler kinetics than the Sucrose BM23 model, have medium high condition numbers (around 10^5^–10^6^).

When the perturbation level increases from 0, there is a clear abrupt condition number change around 30–60% perturbation amplitude. This value varies among the models, in detail, 50% for Sucrose PGM model, 60% for Sucrose BM23, 30% for Glycolysis BM42, and 55% for Signaling BM66. After this perturbation level, all the models turn to ill-posed problems with very high condition numbers.

### Goodness of the Reverse Jacobian upon Covariance Perturbations

**Figure 3:**
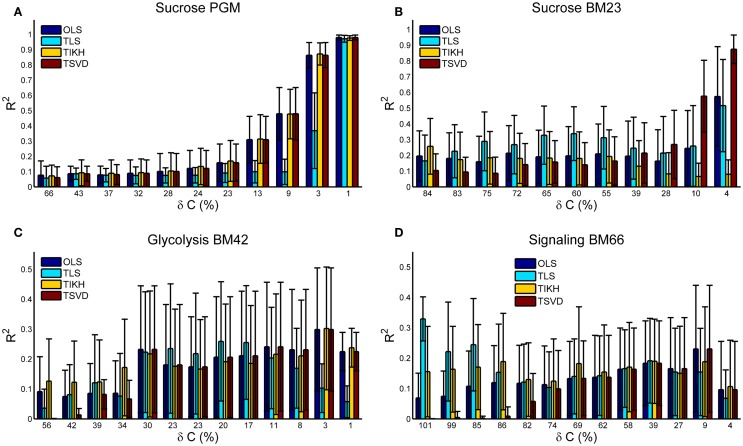
**The goodness of the reverse Jacobian obtained by OLS, TLS, TIKH, and TSVD is represented by the *R*^2^ values when regressed to the true Jacobian (vectorized, see Introduction)**. **(A)** is for Sucrose PGM model, **(B)** for Sucrose BM23 model, **(C)** for Glycolysis BM42 model, and **(D)** for Signaling BM66 model. In each sub figure, the error bar with 1 SD is plotted from 100 iterations. Abbreviation: OLS, ordinary least squares; TLS, total least squares; TIKH, Tickhonov regularization; TSVD, truncated singular value decomposition.

Under no or small covariance perturbations (δ*C* ≤ 10%), the reverse Jacobian calculated by OLS and TSVD shows a high accuracy with *R*^2^ > 0.9 for Sucrose PGM and Sucrose BM23 model. OLS and TSVD are exactly the same for models with small condition number including Sucrose PGM, BM42, and BM66 (Figures 2 and 3A,C,D). For the model with large condition number, TSVD is significantly better than OLS (Mann–Whitney *U* test *p*-value < 1e−11), as observed on BM23 model (Figures 2 and 3B). Under the medium perturbation (30% > δ*C* > 10%), TSVD accuracy drops (*R*^2^ around 0.3) but is still better or similar compared to other methods (Figures 3A,C,D), while OLS drops more than TSVD (Figure 3B). When the perturbation gets larger (δ*C* ≥ 30%), TSVD and OLS accuracies drop drastically and are exceeded by TLS or TIKH. It is also observed that when the perturbation gets larger, the covariance *C* tends to be not positive definite and close to singular, which makes the condition number of *A* very large and thus ill-conditioned (Table S1 in Supplementary Material).

Total least squares (TLS) appears to perform better under large covariance perturbations. This is consistent with its principle (see Introduction and Eq. 8) as it takes into account the error in the covariance. It is more interesting to see that TLS performs better under medium (Figures 3B,C) to large (Figure 3D) perturbations than it does under small perturbations. This is not surprising though. The accuracy of the reverse Jacobian depends on the combined effects from these factors: (1) the approximation solution obtained by each method and (2) the amplitude of perturbations on the covariance. TLS shows lower approximation accuracy but a higher robustness against covariance perturbations while TSVD shows higher approximation accuracy and lower robustness against covariance perturbations. Such a combination yields a non-monotonic change pattern of the reverse Jacobian goodness when the perturbation amplitude increases. Similar phenomena are also observed with TIKH curves in Figures 3B,D.

BM42 and BM66 models show a relatively low accuracy of reverse Jacobian even at small perturbations (Figures 3C,D). One reason may be partly due to their medium-to-high condition number (Figure 2). The other reason may be attributed to the “stiffness” of the system, which is reflected in the Jacobian entries that some entries are many magnitudes larger than others. This yields problems in both solving overdetermined systems and *R*^2^ calculation. To estimate such stiffness, we calculated the ratio between maximal and minimal absolute values of non-zero Jacobian entries, and found that these ratios for BM42 and BM66 are much bigger than in the other two models. The ratio is Sucrose PGM, 388; Sucrose BM23, 3192; Glycolysis BM42, 1.3e6; and Signaling BM66, 1.0e6.

### Goodness of the Reverse Jacobian upon Fluctuation Matrix Perturbations

We investigated the effects of perturbations on the fluctuation matrix *D* over the reverse Jacobian. Since we found the effects for all the models are similar, here we present the results for the Sucrose PGM model. The perturbation levels δ*D* are controlled by adding randomness to the original fluctuation matrix as described in Section “MATERIALS AND METHODS.” The levels are approximately at three scales: 2, 20, and 100%. Here we only investigate the fluctuation matrix perturbation effects, and leave combined effects from both covariance and fluctuation matrices perturbation in the later section. All the models used in this study correspond to the same (and small) covariance perturbation levels, which are the same as the ones of the first bar in Figure 3A.

**Figure 4:**
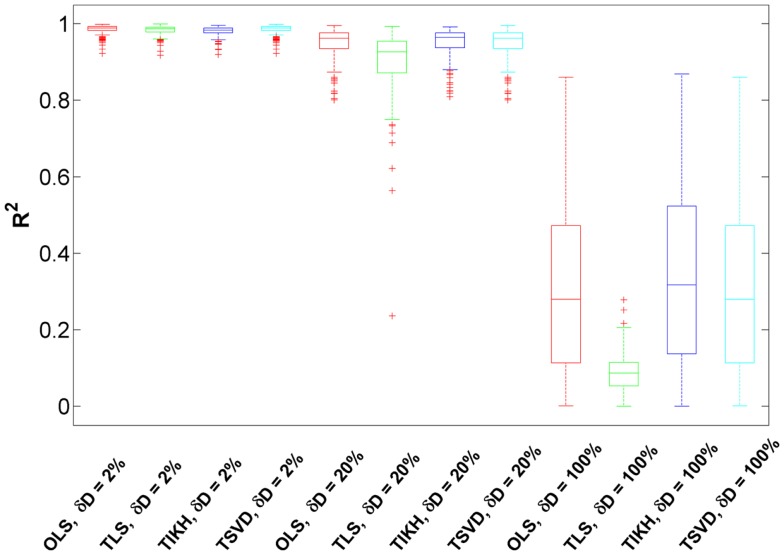
**The goodness of the reverse Jacobian for the Sucrose PGM model calculated from four methods under three levels of fluctuation matrix perturbations is represented as *R*^2^ when regressed to the true Jacobian (vectorized, see Introduction)**. The perturbation is represented as relative change δD with three levels, 2, 20, and 100%. The four reverse calculation methods are OLS, ordinary least squares; TLS, total least squares; TIKH, Tikhonov regularization; and TSVD, truncated singular value decomposition. Each boxplot shows the distribution of *R*^2^ over 100 iterations.

We found that for small-to-medium fluctuation matrix perturbations (δ*D* = 2–20%), the reverse Jacobian has a high accuracy indicated by *R*^2^ which are generally over 0.90 for all reverse calculation methods (Figure 4). Compared to the ones without fluctuation matrix perturbations in the previous section (Figure 3A), the reverse Jacobian accuracies are almost not affected, indicating the additive small randomness on the fluctuation matrix has little effect on the solution.

When the perturbation level increases to 100% and the fluctuation matrix turns to be fully randomized, the reverse Jacobian drops significantly (Figure 4, *R*^2^ centering around 0.3 and ranging from 0 to 0.8). TLS shows the largest drop, indicating it is more sensitive to fluctuation perturbations. For other methods (OLS, TIKH, and TSVD), although more than 75% of *R*^2^ are below 0.6, some *R*^2^ are as high as 0.8. It indicates that there is a possibility to achieve a good reverse Jacobian under some unknown conditions without knowing the fluctuation matrix at all. However, this needs to be further investigated.

### Goodness of the Reverse Jacobian upon Perturbations on Both Covariance and Fluctuation Matrices

**Figure 5:**
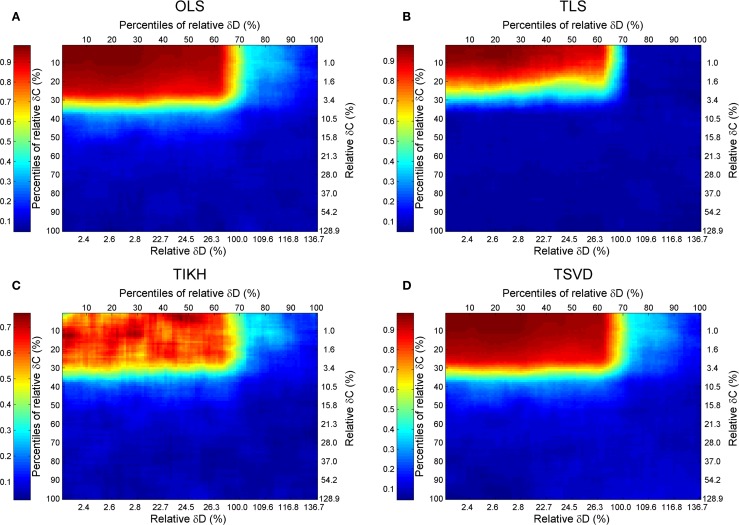
**The goodness of reverse Jacobian for the Sucrose PGM model under perturbations on both covariance and fluctuation matrices is represented as *R*^2^ when regressed to the true Jacobian (vectorized, see Introduction) and shown by the heat map**. The perturbations are measured by the percentile relative and relative changes of δ*C* and δ*D* over covariance and fluctuation matrix, respectively. The percentile relative change is calculated as percentiles of all relative changes δ*C* (or δ*D*). The four reverse calculation methods are **(A)** OLS, ordinary least squares; **(B)** TLS, total least squares; **(C)** TIKH, Tikhonov regularization; and **(D)** TSVD, truncated singular value decomposition. The mean values of all 100 repeats for each level of perturbations are plotted.

Combining the previous results, we give a full map of the combined effect of perturbations on both covariance and fluctuation matrices with the Sucrose PGM model (Figure 5). A general pattern of the combined effects is that the accuracy of reverse *J* is increasing with decreasing levels of perturbations on *C* and *D*, and the high accuracy border (*R*^2^ ≥ 0.7) lies around 30% *C* and *D* perturbation, except the TIKH method where there are a few non-monotonic changing area (Figure 5C). The high accuracy border looks as a mirrored L-shape.

For this specified Sucrose PGM model, being its low condition number, the OLS and TSVD produce similar patterns with large high accuracy borders (Figures 5A,B). Comparing between TLS and TIKH, the former’s border is smaller yet achieves higher accuracy in small perturbations on *C* and *D* (Figures 5C,D).

## Conclusion

Understanding the regulatory mechanisms of metabolic networks is a challenging yet essential task in current biochemical studies. We previously established a reverse Jacobian reconstruction algorithm to infer the regulation of the metabolic network directly from the covariance data (Sun and Weckwerth, 2012; Nägele et al., 2014). In this study; we explored and evaluated the performance of several inverse calculation methods, including OLS, TLS, Tikhonov regularization (TIKH), and TSVD, under the conditions of erroneous covariance and uncertain fluctuation matrix. We simulated four *in silico* representative kinetic models of different levels of complexity with SDEs and obtained the *in silico* data.

We benchmarked these four inverse calculation methods under small-to-large perturbations on the covariance and fluctuation matrices. We found that the accuracy of reverse Jacobian is dependent on these factors: (1) the condition number of *A* in the linear form of Lyapunov Equation as *Ax* = *b*, (2) the perturbation amplitude of covariance, and (3) the stiffness of the kinetic models. The perturbation on the fluctuation matrix, however, has less effect on the reverse Jacobian. A good reverse Jacobian can be obtained with small covariance perturbations and small to medium fluctuation matrix perturbations. Although very few, there are some cases under large covariance and fluctuation matrix perturbations where the reverse Jacobians are similar to their true form. The overall combined effects from covariance and fluctuation matrix perturbations yields a mirrored L-shaped curve.

Tested on the four models, TSVD has achieved highest reverse Jacobian accuracy. OLS performs well when both the condition number of *A* and the perturbation levels are small, but its performance drops down quickly if these conditions are not satisfied. TLS shows robustness against perturbations on the covariance matrix but displays sensitivity to perturbations on the fluctuation matrix. TIKH has similar robustness as TLS upon covariance perturbations and shows less sensitive to fluctuation matrix perturbations.

By systematically comparing inverse calculation methods on systems with inherent error or uncertainties, our study contributes not only to solving Jacobian from metabolomics covariance data, but also to solving ill-posed inverse problems widely studied in many other sciences.

## Conflict of Interest Statement

The authors declare that the research was conducted in the absence of any commercial or financial relationships that could be construed as a potential conflict of interest.
