# Supplementary figures and images for: Challenges of Inversely Estimating Jacobian from Metabolomics Data
Source: Front Bioeng Biotechnol. 2015 Nov 18;3:188. doi: 10.3389/fbioe.2015.00188 (PMC4649029; doi:10.3389/fbioe.2015.00188)

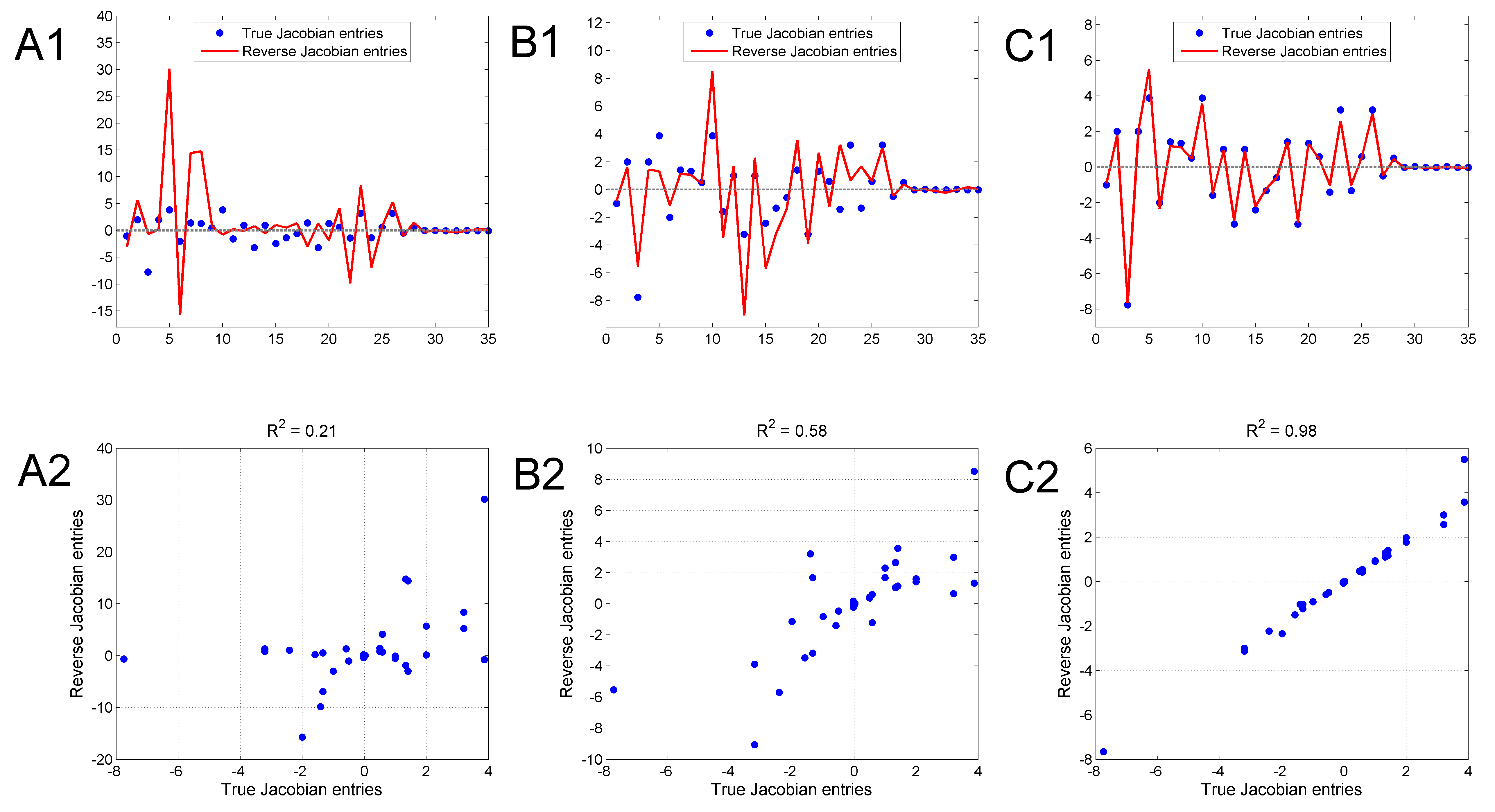

Supplement: Supplementary file 1 [file image_1.png]

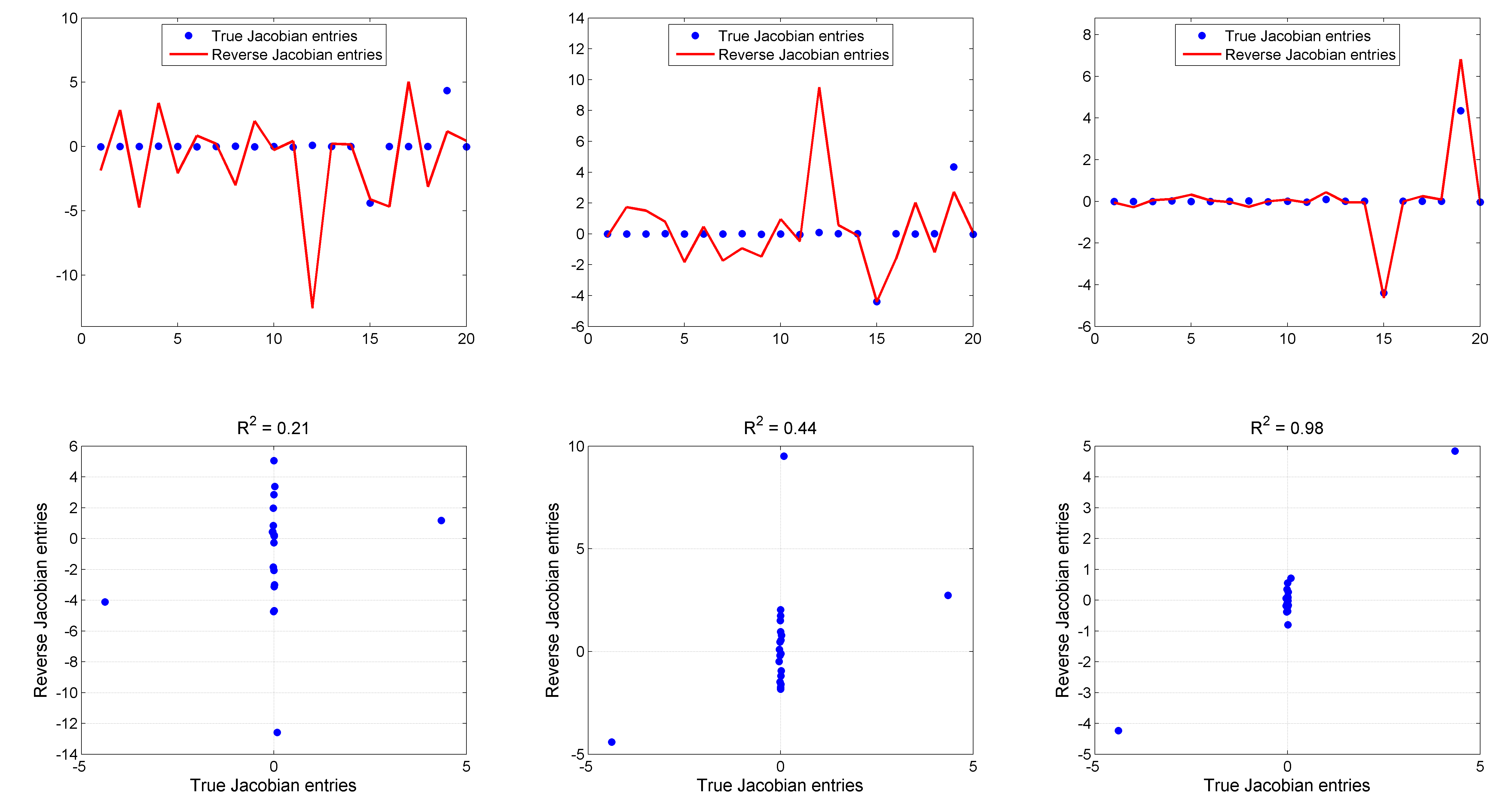

Supplement: Supplementary file 2 [file image_2.png]

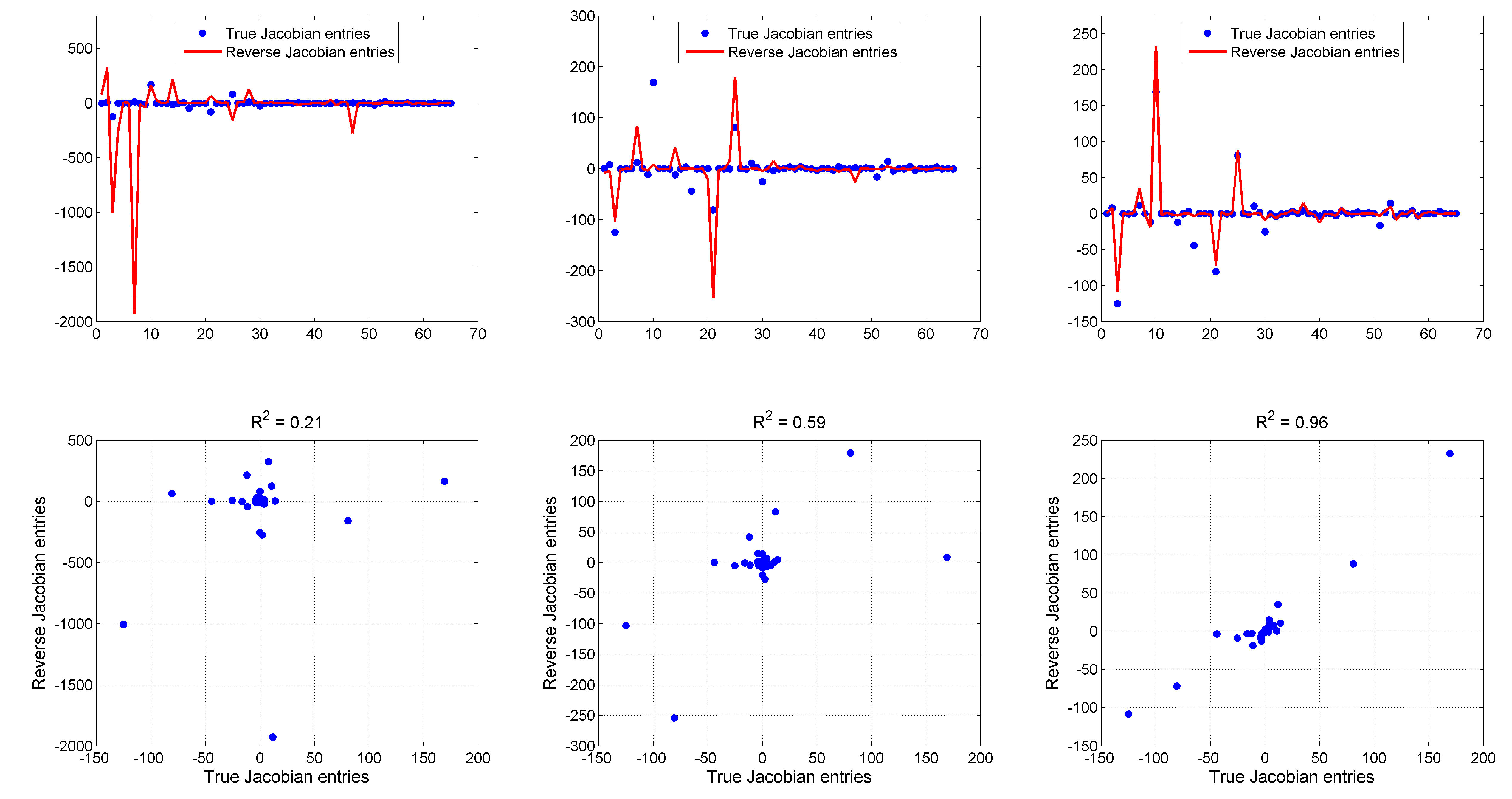

Supplement: Supplementary file 3 [file image_3.png]

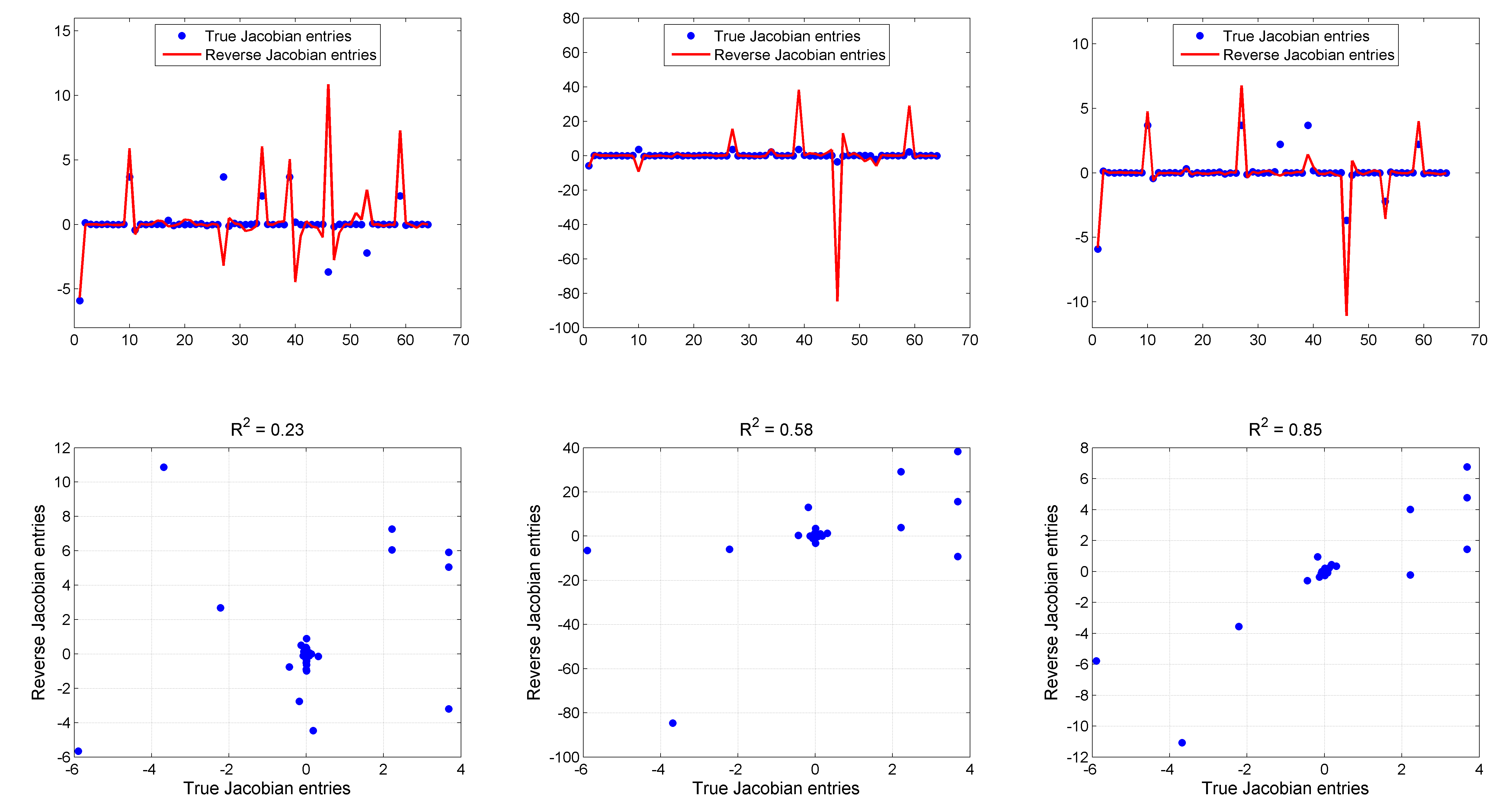

Supplement: Supplementary file 4 [file image_4.png]
