# Supplementary material for: Challenges of Inversely Estimating Jacobian from Metabolomics Data
Source: Front Bioeng Biotechnol. 2015 Nov 18;3:188. doi: 10.3389/fbioe.2015.00188 (PMC4649029; doi:10.3389/fbioe.2015.00188)
Supplement: Supplementary file 5 [file data_sheet_1.pdf]

Challenges of inversely estimating Jacobian from metabolomics data

Xiaoliang Sun[1,2,*], Bettina Länger[1, a], Wolfram Weckwerth[1]

[1]: Department of Ecogenomics and Systems Biology, University of Vienna

[2]: Institute of Integrative Biology, University of Liverpool

[a]: Current address: Siemens AG Austria, Vienna

[*]: correspondence to Xiaoliang Sun, xiaoliang.sun@univie.ac.at

Supplementary Materials:

Figure S1-4. Comparison between true and reverse Jacobian entries in Sucrose PGM model (Figure S1), BM23 model (Figure S2), BM42 model (Figure S3) and BM66 model (Figure S4). We use Figure S1 to explain the captions and legends as they are the same for all other figures. Sub-figure A1, B1 and C1 plot the true entries (blue dots) and reverse entries (red lines) along their locations in the Jacobian matrix (altogether 35 non-zero entries in the Jacobian) Sub-figure A2, B2 and C2 show the pairwise comparisons between true and reverse entries. A, B and C denote three ranges of R^2 (Low: A, [0.2, 0.3]; Medium: B, [0.4, 0.6]; High: C, [0.85, 1]).

Table S1. The relation between property of covariance *C* and the condition number κ of the Sucrose PGM model under different level of perturbations on C. Negative eigenvalues of *C* indicates that *C* is not positive definite and thus Lyapunov function* VCV' is not positive too, where V is called Lyapunov candidate function. (*: Keller-Ressel, Martin. "Lyapunov Function." From MathWorld--A Wolfram Web Resource, created by Eric W. Weisstein. http://mathworld.wolfram.com/LyapunovFunction.html)

Relative change of *C* (%)	Positive Definite?	Minimal Eigenvalue	Maximal Eigenvalue	Condition number κ
65.75	No	-5.36E-19	0.012	3.00E+18
52.57	No	-5.98E-19	0.037	6.99E+17
47.60	No	-7.15E-18	0.13	1.97E+17
42.95	No	-3.15E-18	0.015	2.09E+08
36.76	No	-1.80E-19	0.061	1968859
31.88	No	-2.02E-19	0.099	26728.68
27.94	Yes	4.60E-11	0.065	10698.63
23.43	Yes	4.61E-09	0.10	6455.59
22.12	Yes	3.68E-08	0.037	5443.70
12.61	Yes	2.77E-06	0.025	5092.06
8.27	Yes	4.64E-06	0.042	5655.65
2.87	Yes	5.97E-06	0.05	8640.71
0.95	Yes	5.76E-06	0.056	10815.87
